# Dynamic Versus Stable Fixation of Distal Tibiofibular Joint Injuries: A Meta-Analysis Comparing Functional Outcomes

**DOI:** 10.7759/cureus.110680

**Published:** 2026-06-11

**Authors:** Leon Liu, Pradith Santapur, Taylor Checkley, Andy Suarez, James Ro, Gary Schwartz

**Affiliations:** 1 Allopathic Medicine, Nova Southeastern University, Dr. Kiran C. Patel College of Allopathic Medicine, Fort Lauderdale, USA; 2 Orthopedic Surgery, Nova Southeastern University, Dr. Kiran C. Patel College of Allopathic Medicine, Fort Lauderdale, USA

**Keywords:** dynamic fixation, dynamic single screw fixation, stable fixation, tibio-fibular joint injury, tibio-fibular syndesmosis

## Abstract

Syndesmotic injuries are associated with chronic instability, persistent pain, and post-traumatic osteoarthritis. Management typically involves either static fixation with screws or dynamic fixation with suture button devices. While static fixation has historically been the standard of care, dynamic fixation allows for the preservation of physiological joint micromotion at the distal tibiofibular syndesmosis and may thus improve functional outcomes.

A systematic review and meta-analysis was conducted in June 2025 using only PubMed and Google Scholar and no additional databases. Only prospective randomized controlled trials (RCTs) involving adult patients with syndesmotic injuries were included. Four RCTs comprising 250 patients were ultimately selected. Data were extracted using Covidence, risk of bias was assessed with RevMan 5.4.1, and pooled outcomes were analyzed using a random-effects model. Functional outcomes were measured using the American Orthopaedic Foot and Ankle Society (AOFAS) score and the Olerud-Molander Ankle Score (OMAS); the Visual Analogue Scale (VAS) was used to measure and compare postoperative pain.

Dynamic fixation demonstrated comparable AOFAS scores (mean difference = 3.14; 95% CI: -5.34 to 11.62; p = 0.47, I² = 96%) and significantly higher OMAS scores (mean difference = 5.70; 95% CI: 0.33-11.08, p = 0.04, I² = 79%) compared with static fixation, though clinically meaningful thresholds were not met. Patients treated with dynamic fixation also reported lower pain scores (VAS mean difference = -0.77; 95% CI: -1.27 to -0.27; p = 0.003, I² = 52%). Risk of bias was low to moderate, with limitations primarily related to incomplete blinding.

Dynamic fixation for syndesmotic injuries is associated with superior functional outcomes and reduced postoperative pain compared to static screw fixation. However, the substantial heterogeneity observed across pooled estimates, particularly for AOFAS (I² = 96%), limits confidence in these findings and precludes definitive conclusions. Larger RCTs with standardized postoperative protocols and reduced methodological variability are needed before guideline recommendations can be made.

## Introduction and background

Distal tibiofibular joint injuries are characterized by disruption of the ligamentous complex that acts to stabilize the distal articulation between the tibia and fibula [1,2]. Also referred to as syndesmotic injuries, these injuries have an annual incidence of 15 per 100,000 individuals and occur concomitantly with ankle fractures in 1-11% of cases [3,4]. 

The syndesmosis is stabilized by a complex of four ligaments, including the anterior inferior tibiofibular ligament (AITFL), the posterior inferior tibiofibular ligament (PITFL), the transverse tibiofibular ligament (TTFL), and the interosseous ligament (IOL). The deltoid ligament serves as the primary medial stabilizer of the ankle joint [1,2]. Together, these structures allow for a small but physiologically important range of motion at the distal tibiofibular joint, which is essential to normal ankle biomechanics [2,5]. Thus, disruption of this ligamentous complex leads to joint destabilization and alters the natural micromotion that any surgical fixation strategy must account for. These injuries are also associated with significant sequelae such as chronic instability, persistent pain, and trauma-induced osteoarthritis, making proper management particularly important [1].

Syndesmotic injuries occur across a spectrum of pathology. Classification of these injuries follows a similar paradigm to that of ankle sprains, quantified by the level of ligamentous injury. Grade I injuries are stretch injuries, grade II are partial tears, and grade III injuries are characterized by a complete tear of the syndesmosis [1,2]. Fracture-associated syndesmotic injuries are incorporated into the Weber classification of lateral malleolus fractures [1,2]. Weber A fractures are characterized by a preserved syndesmosis, while type B fractures have a variable incidence of injury (11-39%) [1]. Lastly, Weber C fractures are, by definition, associated with a syndesmotic injury, as they occur at the level of the syndesmosis [2].

Surgical treatment options for distal tibiofibular joint injuries involve management with either static or dynamic fixation techniques [6]. The traditional standard, static fixation, involves the usage of transsyndesmotic screws to achieve rigid stabilization of the syndesmosis and prevent movement of the tibia and fibula [6,7]. Despite its widespread usage, this method is associated with several postoperative complications, including screw breakage, risk of malreduction, and the necessity of a hardware removal procedure [6-9]. One paper reported 22% of static fixation surgeries requiring reoperation and roughly 10% suffering an infection within one year after the procedure, while another reported reoperation rates of almost 40% [10].

Dynamic fixation was named for its facilitation of physiological motion at the syndesmosis to better imitate the natural biomechanics of the distal tibiofibular joint [1,11]. Its design was intended to permit earlier weight-bearing, eliminate the need for secondary operations, and reduce complication rates [11,12]. Early research indicates that dynamic fixation may yield comparable functional outcomes when contrasted with the standard static fixation method [12].

Dynamic fixation is typically performed with the suture button technique [13]. This involves using metallic or polymer buttons to secure a single suture passed through the tibia and fibula, maintaining reduction of the injury while allowing a small degree of motion [12,13]. Compared to static fixation, dynamic fixation is associated with a lower reoperation rate of between 7.7% and 10.5% [8,10].

Comparisons of the fixation strategies and their postoperative complication rates are prevalent in the literature; however, there remains discourse in the most optimal approach of stabilizing syndesmotic injuries. For example, meta-analyses have identified lower complication and reoperation rates associated with dynamic fixation and, in some analyses, improved early functional outcomes compared to static fixation, but the magnitude and consistency of these findings vary across studies [8,11,14,15]. Although dynamic fixation has demonstrated biomechanical advantages over screw fixation, it remains unclear whether these advantages translate into clinically meaningful improvements in post-op functional outcomes and reductions in quality. This systematic review and meta-analysis aimed to evaluate functional outcomes and post-treatment complications comparing static and dynamic fixation for distal tibiofibular joint injuries.

## Review

Methods

Study Design and Search Strategy

In June 2025, a systematic search was conducted through PubMed and Google Scholar for literature comparing the functional outcomes of patients treated with dynamic and stable fixation. Search terms were developed based on concepts related to syndesmotic injury and surgical fixation techniques. The exact search syntax is listed below:

Pubmed search syntax: (“syndesmosis” OR “syndesmotic injury” "ankle syndesmosis" OR "distal tibiofibular joint" OR “distal tibiofibular”[All Fields]) AND "injury"[All Fields] AND ("dynamic fixation"[All Fields] OR "static fixation"[All Fields] OR "suture button"[All Fields] OR "screw fixation"[All Fields]) AND "comparing"[All Fields]

The following MeSH terms were also applied to the PubMed search: adult, human, bone screws, prospective studies, and treatment outcomes. 

Google Scholar search syntax: "ankle syndesmosis" AND ("dynamic fixation" OR "suture button") AND ("syndesmotic screw" OR "static fixation") AND ("randomized controlled trial" OR prospective). 

No publication date restrictions were applied, and only English-language studies were eligible, returning a total of 1,263 results.

Study Selection

The following Population, Intervention, Comparison, and Outcome (PICO) question was addressed: In adult patients with isolated ankle syndesmotic injuries and fracture-associated syndesmotic injuries requiring operative repair, how does dynamic fixation compare with static fixation in terms of postoperative functional outcomes? Selection criteria included randomized controlled trials written in English. Studies were eligible for inclusion if they were conducted on adult patients with either isolated syndesmotic ligamentous injuries or fracture-associated injuries. When outcomes were not stratified by injury type, they were included together in a pooled analysis and considered as a source of heterogeneity. "Dynamic fixation" was defined as either TightRope/suture-button or Flexible/dynamic syndesmotic fixation devices. "Stable fixation" was defined as syndesmotic screw fixation and metallic screw fixation for the purposes of this analysis. Eligible studies were also selected if patients were followed at least one year postoperatively, and outcome measurements were quantified via the American Orthopaedic Foot & Ankle Society (AOFAS) [16] score, Olerud-Molander Ankle Score (OMAS) [17], and Visual Analogue Scale (VAS) for pain. 

**Figure 1:**
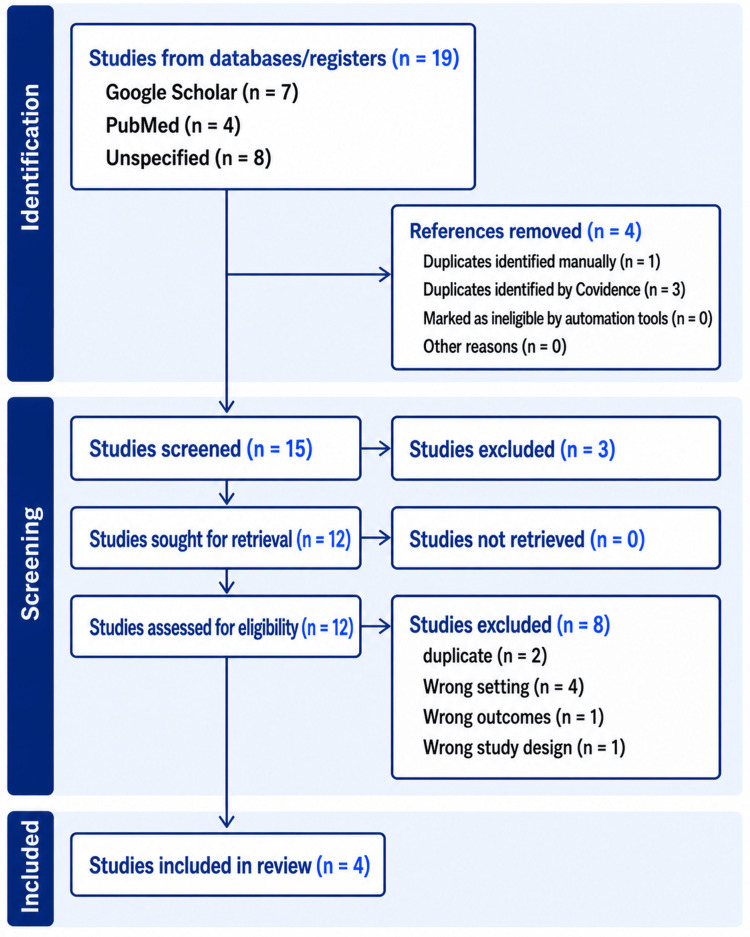
PRIMSA flowchart for this meta-analysis. PRISMA: Preferred Reporting Items for Systematic Reviews and Meta-Analyses.

Studies published in languages other than English were removed, along with duplicate patient cohorts and formats such as case reports, retrospective studies, cadaver studies, animal studies, and systematic reviews. No time limits or additional filters were applied. The inclusion criteria designated four prospective randomized controlled trials that were used in this review following the Preferred Reporting Items for Systematic Reviews and Meta-Analyses (PRISMA) protocol [18] (Figure 1). The protocol was submitted to PROSPERO and is currently awaiting approval.

Following the literature search, the studies were uploaded to Covidence (Covidence systematic review software, Veritas Health Innovation, Melbourne, Australia) [19]. The data extraction tool was used, and duplicates were automatically excluded. Two independent reviewers screened the articles based on title and abstract. Studies that did not include the outcomes of interest or follow the appropriate study design were excluded. Remaining studies underwent full-text screening by two independent reviewers using the predetermined inclusion and exclusion criteria. Each reviewer worked independently, and article selection was blinded. Discrepancies in article selection were resolved through discussion with a third reviewer.

Data Extraction and Risk of Bias

Data extraction was performed by two independent reviewers utilizing templates provided by Covidence [19]. The templates were used to collect data regarding the background of the article, methods used, population characteristics, interventions, and outcomes. Some studies also reported outcomes specific to their unique research objectives; however, those outcomes were not included in this meta-analysis. 

Risk of Bias was assessed using the Cochrane Risk of Bias tool RoB 2 [20]. Various parameters of each study were scored by two individual reviewers using a scale of low to high likelihood of bias. Parameters scored involved aspects of study design, such as blinding, selective reporting, and random sequence generation.

Data Analysis

Functional outcomes were primarily analyzed; these were gauged with several metrics, including the American Orthopaedic Foot & Ankle Society (AOFAS) [16] score, the Olerud-Molander Ankle Score (OMAS) [17], and the Visual Analogue Scale (VAS) for pain. Extracted data were pooled and analyzed using the Review Manager (RevMan) Tool Version 5.4.1 (The Cochrane Collaboration, London, United Kingdom, 2020). Mean differences were estimated, and the inverse variance statistical method was applied. A random-effects model was applied, given anticipated data heterogeneity, and point estimates were calculated with their corresponding 95% confidence intervals (CIs). All tests were given a statistical significance threshold of 0.05 and heterogeneity was assessed by calculating the I² index with values >50% representing moderate to high heterogeneity. 

Results 

The initial search query returned 19 studies that underwent abstract and title screening. After the resulting studies underwent full-text screening, a total of four studies [21-24] were eligible for inclusion in the final data analysis of this meta-analysis. Outlined below are the baseline characteristics of the selected studies (Table 1).

**Table 1:** Baseline characteristics of included studies. LOE: level of evidence, NR: not reported. *Group-specific age was not reported; value represents the overall cohort. References: [21-24].

Name, year	Country	Design	LOE	Participants (n)	Stable fixation age, y	Dynamic fixation age, y	Male/female (%)
Andersen et al., 2018	Norway	RCT	I	97; Stable fixation: 49; Dynamic fixation: 48	43.0 ± 16.2	46 ± 14.8	66/34
Gungor et al., 2024	Türkiye	RCT	NR	48; Stable fixation: 24; Dynamic fixation: 24	37.3±15.1*	NR	58/42
Kortekangas et al., 2015	Finland	RCT	I	40; Stable fixation: 19; Dynamic fixation: 21	43.0 ± 15.7	46 ± 14.8	63/37
Laflamme et al., 2015	Canada/ Netherlands	RCT	II	70; Stable fixation: 36; Dynamic fixation: 34	39.3 ± 12.4	40.1 ± 13.8	73/27

Outcomes

**Figure 2:**
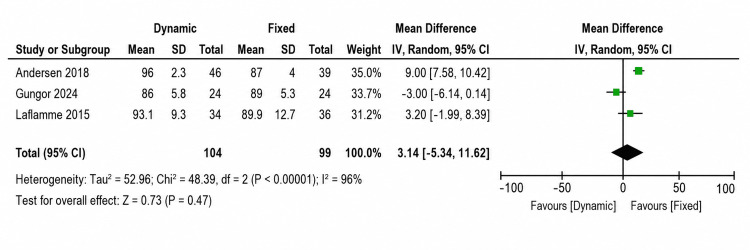
AOFAS outcome analysis. AOFAS: American Orthopaedic Foot & Ankle Society. References: [21,22,24].

The analyses of the data pooled from 246 total patients in the four included studies demonstrated favorable functional outcomes with the dynamic fixation technique. AOFAS scores were higher with dynamic fixation but not statistically significant (mean difference = 3.14; 95% CI: -5.34 to 11.62; p = 0.47 6.74; 95% CI: 5.49-8.00; p = 0.47) across three studies, including 203 patients (Figure 2). Additionally, heterogeneity was notable (I² = 96%), indicating substantial variability across studies.

**Figure 3:**
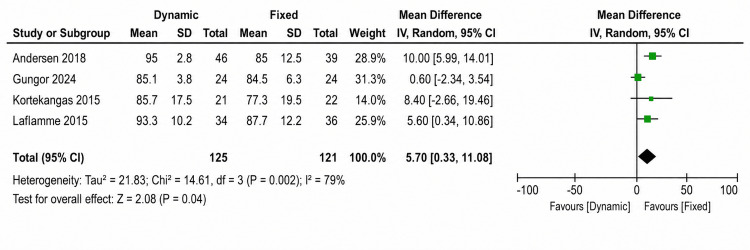
OMAS outcome analysis. OMAS: Olerud-Molander Ankle Score. References: [21-24].

The OMAS indicated superior functional scores in the dynamic fixation group (mean difference = 5.70, 95% CI: 0.33 to 11.08; p = 0.04) across four studies, including 246 patients (Figure 3). This metric was associated with moderate-to-high heterogeneity (I² = 79%).

**Figure 4:**
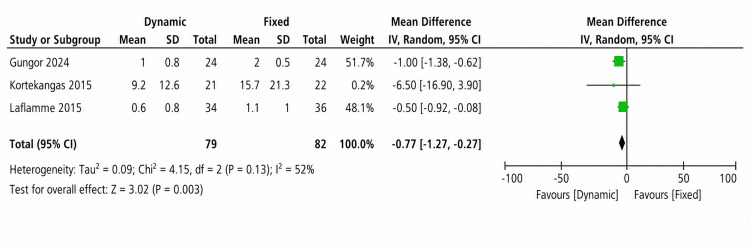
VAS pain score analysis. VAS: Visual Analogue Scale. References: [21-23].

The VAS was used to assess pain in three studies with a total of 161 patients. Patients in the dynamic fixation group reported lower pain scores (mean difference = -0.77, 95% CI: -1.27 to -0.27; p = 0.003) than those treated with stable fixation (Figure 4). Heterogeneity was moderate for this analysis (I² = 52%).

Risk of Bias

**Figure 5:**
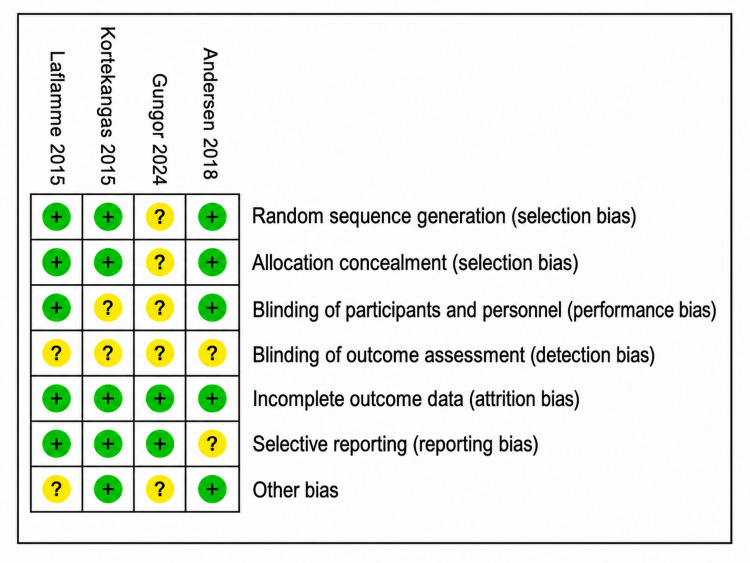
Risk of bias summary. References: [21-24].

**Figure 6:**
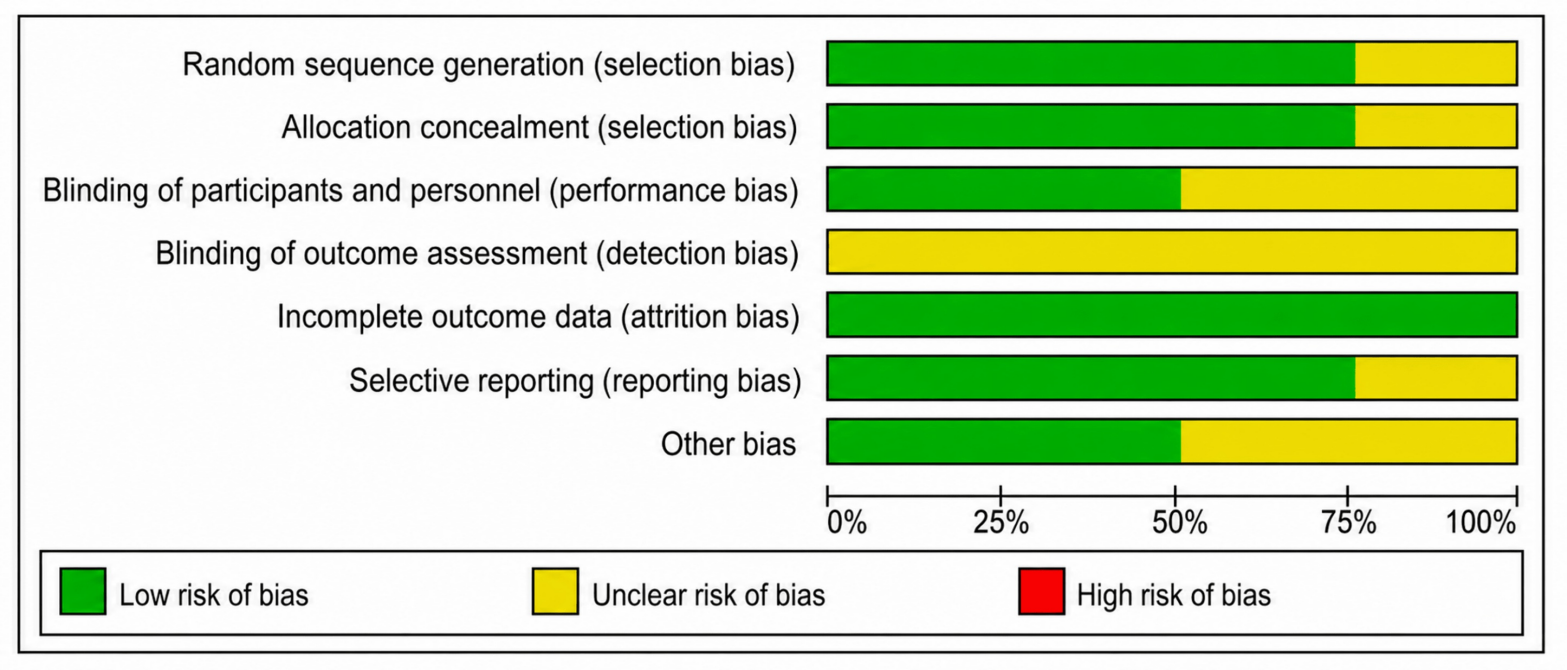
Risk of bias graph. References: [21-24].

The risk of bias was assessed for all four studies. Bias primarily came from incomplete blinding of participants and surgical staff, consistent with the methodology and hypothesis of the studies included. A few articles also had unclear bias in the blinding of outcome assessment, allocation concealment, and completion of outcome data (Figures 5, 6).

Discussion

Among patients evaluated in this meta-analysis, OMAS functional outcome metrics demonstrated that dynamic fixation was superior and pain measurements were marginally lower. Despite demonstrating statistically significant improvement, the observed mean differences of AOFAS and OMAS scores (3.14 and 5.70 points, respectively) fall below the commonly accepted threshold for a clinically meaningful difference for each score, raising the question of whether these findings represent clinically meaningful benefit [6]. However, given the statistical significance, dynamic fixation remains a favorable option in addressing syndesmotic injuries.

There are several important considerations when evaluating the utility of dynamic fixation. As seen in Xu et al. [25], the recovery process with dynamic fixation may also differ from that of screw fixation. That study reported a quicker return to weight-bearing and higher functional measurements in short-term follow-up. Though this study in particular was limited, given the smaller sample size and shorter follow-up, it does provide insight into the immediate recovery following dynamic fixation. Malreduction rates have also been suggested to be comparable and sometimes lower in dynamic fixation, indicating its structural efficacy [23]. Additional advantages of dynamic fixation include the retention of physiologic joint motion [1,11] as well as eliminating the need for a second procedure for hardware removal [6,7,22]. These findings, in combination with higher functional outcomes, suggest that dynamic fixation has additional benefits in its use.

Based on this meta-analysis, dynamic fixation is the recommended treatment option to address syndesmotic injuries. With quick recovery, stable reduction, and superior outcomes, patients may benefit from this technique when compared to its alternative. One possible concern with dynamic fixation is that its benefits may be offset by the increased initial cost; however, the overall cost of dynamic fixation has been shown to be lower when there is a necessity for hardware removal, as seen in stable screw fixation [26].

High heterogeneity was observed in the analysis of AOFAS data. Among the included studies, high variance in the AOFAS score may have been due to variation of surgical technique, surgical hardware, and injury classifications among patients. Moreover, variations in postoperative rehabilitation protocols, such as follow-up length, differences in weight-bearing timelines, and the volume and intensity of physiotherapy, may have contributed to data variability across studies. The aforementioned variability in both the AOFAS and OMAS scores may restrict the interpretations drawn from this data set. Additional limitations of this study include the small overall sample size (250 patients), which remains a common challenge in orthopedic literature, and the reliance on PubMed and Google Scholar as the primary sources for the literature search. Although these sources yielded a large number of results, the exclusion of additional databases may have increased the risk of incomplete retrieval of eligible studies.

Despite its limitations, this meta-analysis also presents a strong case for its recommendations. With only the inclusion of randomized controlled trials as well as a rigorous selection criteria with multiple independent reviewers, risks related to selection bias and study selection are minimized. Using established outcome measurements in the AOFAS, OMAS, and VAS scores provides a standardized platform to compare data across studies. Ultimately, future studies will require larger randomized controlled trials with standardized postoperative protocols to draw stronger conclusions. Standardizing surgical techniques, hardware used, and metrics for assessing postoperative complication rates will yield a stronger data pool. Though this meta-analysis supports the use of dynamic fixation techniques in syndesmotic injuries, additional research must be conducted before guideline recommendations are made.

## Conclusions

Dynamic fixation for syndesmotic injuries may provide better functional outcomes and less postoperative pain when compared with static screw fixation. Superior outcomes in conjunction with comparable joint reduction present a case for dynamic fixation as a favorable option when treating ankle syndesmotic injuries in the adult population. Although increased heterogeneity among studies in the current meta-analysis prevents definitive conclusions regarding a single preferred fixation method, the findings support the continued use of dynamic fixation as a clinically effective option. Further randomized controlled trials with standardized outcome measures are required to aid in treatment selection for these injuries.
